# Evaluation of the usage of incisional liposomal bupivacaine as a local anaesthetic for dogs undergoing limb amputation

**DOI:** 10.1002/vms3.1159

**Published:** 2023-06-06

**Authors:** Ashley S Villatoro, Holly A Phelps, Justin B Ganjei

**Affiliations:** ^1^ Veterinary Surgical Centers Vienna Austria; ^2^ Veterinary Surgical Centers Leesburg Virginia USA; ^3^ Veterinary Referral Associates Vienna Virginia USA

**Keywords:** control group (CG), liposomal bupivacaine group (LBG), liposomal bupivacaine suspension (LBS)

## Abstract

**Background:**

Bupivacaine lioposomal suspension has recently emerged in the veterinary field for local analgesia.

**Objective:**

To describe the extra‐label administration of bupivacaine liposomal suspension at the incision site of dogs undergoing limb amputation and characterize any complications.

**Study Design:**

Nonblinded retrospective study.

**Animals:**

Client‐owned dogs undergoing limb amputation from 2016 to 2020.

**Methods:**

Medical records of dogs undergoing limb amputation with concurrent use of long‐acting liposomal bupivacaine suspension were reviewed for incisional complications, adverse effects, hospitalization length, and time to alimentation. Data were compared to a control group (CG) of dogs who underwent a limb amputation procedure without concurrent use of liposomal bupivacaine suspension.

**Results:**

Forty‐six dogs were included in the liposomal bupivacaine group (LBG) and 44 cases in the CG. The CG had 15 incidences of incisional complications (34%) compared to 6 within the LBG (13%). Four dogs required revisional surgery in the CG (9%) whereas none of the dogs required revisional surgery in the LBG. Time from surgery to discharge was statistically higher in the CG compared to the LBG (*p* = 0.025). First time to alimentation was statistically higher in the CG (*p* value = 0.0002). The total number of rechecks needed postoperatively revealed the CG having a statistically significant increase in recheck evaluations (*p* = 0.001).

**Conclusions:**

Extra‐label administration of liposomal bupivacaine suspension was well‐tolerated in dogs undergoing limb amputation. Liposomal bupivacaine usage did not increase incisional complication rates and its use allowed for a quicker time to discharge.

**Clinical significance:**

Surgeons should consider inclusion of extra‐label administration of liposomal bupivacaine in analgesic regimens for dogs undergoing limb amputation.

## Graphical abstract

Retrospective study comparing the use of bupivacaine liposomal suspension at the incision site of client owned dogs undergoing limb amputation with a group of dogs that did not receive the drug. The liposomal bupivacaine group had less incisional complications, quicker time to alimentation and discharge, and fewer recheck evaluations. Surgeons should consider inclusion of extra‐label administration of liposomal bupivacaine in analgesic regimens for dogs undergoing limb amputation.

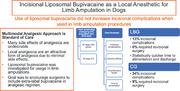

## 1 INTRODUCTION

A multimodal analgesic approach to perioperative pain control has become the standard of care in veterinary orthopaedic surgery. Currently there are many analgesic techniques utilised in dogs undergoing orthopaedic surgery including epidural analgesic administration (Marucio et al., 2014; Odette & Smith, 2013; Smith & Yu, 2001) intraarticular analgesic infusion (Soto et al., 2014; Gurney & Leece, 2014; Dutton et al., 2014), intraperitoneal administration (Costa et al., **2019**), continuous rate intravenous infusion of analgesics (Wagner, 2002; Steagall et al., 2006) and oral analgesic administration (Wagner, 2002; Cardozo et al., 2014; Davila et al., 2013). Regional analgesic methods are a very attractive form of perioperative analgesia, as many of the side effects of systemic opioid analgesics can be avoided or decreased (Carpenter et al., 2004; Wolfe et al., 2006; Abelson et al., 2009; Lemke & Dawson, 2000). Opioids have traditionally been the analgesic class used most commonly for orthopaedic surgery but are known to cause undesirable postoperative side effects such as dysphoria (Becker et al., 2013; Kongara, 2018), gastrointestinal dysfunction such as ileus (Brock et al., 2012; Johnson, 1981), disruption of temperature regulation (Adler et al., 1988) and even adverse immunomodulatory effects (Odunayo et al., 2010). Consequently, efficacious and safe alternatives for postoperative analgesia are continuously sought in veterinary medicine.

A bupivacaine liposomal injectable suspension (LBS) has previously emerged in human medicine as an adjunctive method of multimodal analgesic approach in the postoperative period. Studies comparing LBS to traditional nonliposomal local analgesics have shown superior efficacy of the liposomal bupivacaine in surgery (Barrington et al., 2017; Johnson et al., 2017). A study reviewing LBS used in patients undergoing total knee arthroplasty revealed that its use significantly reduced postoperative pain, opioid consumption, and time to first opioid rescue administration, without any safety concerns (Barnes et al., 2020). A review article of LBS use in plastic and reconstructive surgery revealed it to be efficacious, allowed for a decrease in hospitalisation time, and showed no serious adverse incisional effects with its use (Vyas et al., 2016). In addition, the need for opioid use in the immediate postoperative period was significantly lower than in the control group in people undergoing orthopaedic surgery with no increased risk of wound complications (Robbins et al., 2015).

LBS has been recently introduced into the veterinary field, specifically for postoperative analgesia use in dogs undergoing stifle surgery (Nocita, 2018). Previous studies have shown that dogs undergoing stifle surgery responded favourably to the use of incisional liposomal buivacaine (Reader et al., 2020; Lascelles & Kirkby Shaw, 2016). To the author's knowledge, there are no studies evaluating the use of LBS with orthopaedic procedures outside of stifle surgery, such as limb amputation. A previous study has shown traditional bupivacaine used in conjunction with a wound soaker catheter for limb amputations was associated with good success at lowering pain scores, having minimal to no systemic side effects, and no increase in incidence of incisional infection (Abelson et al., 2009). To the author's knowledge, there are currently no veterinary studies present evaluating the use of subepineural administration of LBS within peripheral nerves for local nerve blockade in dogs.

The purpose of this study was to evaluate the extra‐label use of LBS as a local incisional and subepineural block in dogs undergoing limb amputation and to evaluate for any possible complications. The amount of analgesia required postoperatively and first time to alimentation and hospitalisation length will be investigated concurrently. The authors hypothesise that the use of LBS within incisional closing layers and subepineurally will not be associated with an increase in incidence of incisional complications outside of those previously published.

## 2 MATERIALS AND METHODS

Client owned dogs undergoing a limb amputation procedure with or without concurrent use of long‐acting liposomal bupivacaine suspension (LBS) in hospital were included in the study. Patients that received liposomal bupivacaine were included if between July 2018 and August 2020 (LBG). The time frame was chosen to begin in July 2018 due to addition of liposomal bupivacaine to analgesic regimen at this facility. Patients that did not receive liposomal bupivacaine were included as the control group (CG) if surgery was performed between August 2016 and August 2018, a similar time frame as the liposomal group for similar patient numbers. Patients were searched via hospital issued computer software to include certain keywords (major amputation and/or liposomal bupivacaine; Nocita, Aratana Therapeutics, Leawood, KS). Dogs were included if they had a limb amputation performed without any other concurrent procedure at the time of surgery. The reason for limb amputation was documented along with the breed, sex, age, weight, presenting complaint, presurgical diagnostics, surgeon performing the procedure, surgical site and surgical technique performed, histopathology results (if performed and available).

For those receiving liposomal bupivacaine, the dose of liposomal bupivacaine suspension administered and location injected was documented. For the groups receiving only incisional liposomal bupivacaine suspension, a 5.3 mg/kg dosage of the drug was drawn from the bottle by a surgical assistant. Additional liposomal bupivacaine (35% of cases) or nonliposomal bupivacaine (65% of cases) was drawn up in a 1mL syringe with 25G needle. This amount was first injected in subepineural fashion, proximal to the intended transection site of the isolated nerves directly in the surgical field. Approximately 0.1 to 0.3 mL of liposomal or nonliposomal bupivacaine was used for each nerve depending on size. The previously drawn dosage of liposomal bupivacaine was then injected into tissue layers (muscle, fascia, subcutaneous layers) at the time of incisional closure through a 22G long needle. The needle was inserted to the hub parallel to closing tissue (approximately 0.5–1 cm from the tissue plane) and withdrawn while simultaneously administering. All patients in the control group received subepineural nonliposomal bupivacaine.

Postoperative analgesic medication selections and length of use (if continuous rate infusions) or number of dosages were recorded for each patient. The Colorado State University Canine Acute Pain Assessment was used for pain scoring of the liposomal bupivacaine group only. If any of the recorded values were above the threshold for re‐evaluation for rescue analgesia of ≥2, additional pain relievers were administered by discretion of the attending veterinarian. Time to discharge from the time of surgery; first time to eat and episodes of vomiting, regurgitation or diarrhoea were also recorded for each patient during hospitalisation time.

Hospital and communications records were reviewed for any short‐term postoperative complications within the time from surgery to their final recheck examination (average of 3 weeks postoperatively). Specific postoperative complications assessed included incisional complications such as seroma formation, incisional dehiscence, and incisional infection. If wound infection was present, culture results were also recorded as well as response to treatment. Treatment to any of the above complications was recorded and if any revisional surgery was performed. Revisional surgery was defined as re‐anaesthetising the patient and surgical debridement and closure of the wound.

### 2.1 Statistical analysis

Normally distributed variables were summarised as means (standard deviation), skewed variables as median (range) and categorical variables as counts and percentages. Exposures of interest (LBS vs. CONTROL) were compared using the 2‐sample *t*‐test for normally distributed variables, the Wilcoxon rank‐sum test for skewed variables and Fisher's exact test for categorical variables. Statistical significance was set to *p* < 0.05. All statistical analyses were performed using commercially available software (SAS, version 9.4, SAS institute, Inc, Cary, NC).

## 3 RESULTS

A total of 49 cases were identified in which LBS was used concurrently with a limb amputation procedure. Data were available for all but three of the cases that had incomplete records and were subsequently excluded from the study for a total of 46 cases included in LBG. A total of 44 cases were identified within the CG ranges. The average age of the dogs in the LBG was 8.9 ± 2.7 years (range 2 years to 15 years) and 7.6 ± 2.7 years (range 1.9 years to 12.4 years) for the CG. There was a similar distribution of neutered females and males within both groups. The average weight of the dogs in the LBG was 30.7 ± 13.1 kg (range 3.5 kg to 60.2 kg) and 31.0 ± 15.2 kg (range 3.4 to 66 kg) in the control group. The most common breeds represented were Labrador Retriever (*n* = 19), followed by Greyhounds (*n* = 7), Golden Retrievers (*n* = 7), among a variety of others (Great Dane, Doberman, American Pit Bull, Chesapeake Retriever, Jack Russel Terrier, Pomeranian, Malitpoo).

Normal probability plots showed that age and body weight followed a normal distribution while time to discharge (in hours), time to first alimentation in hospital (in hours), number of postoperative rechecks performed, total length of continuous rate infusions of analgesia (in hours) and number of rescue analgesia required (in number of boluses) were skewed. Categorical variables of interest included if an epidural was administered, if incisional complications were documented postoperatively, if revisional surgery was required following postoperative complications, total length of continuous rate infusions of analgesia (1 vs. 2).

All limb amputation procedures were performed by a board‐certified surgeon. The same group of surgeons operated on the liposomal bupivacaine and control groups with the exception of two surgeons who operated on 12 of the CG patients and none in the LBG. Within the LBG, there were a total of 30 forelimb amputation procedures (scapulectomy) and 16 pelvic limb amputation procedures (11 coxofemoral disarticulation and 5 mid‐femoral). The CG included a total of 20 forelimb amputation procedures (scapulectomy) and 24 pelvic limb amputation procedures (23 coxofemoral disarticulation and 1 mid‐femoral).

The most common reason for limb amputation in the LBG was an aggressive osseous lesion located in the peripheral limb with osteosarcoma being the most common disease process which occurred in 21 of 46 cases (46%). Other disease processes included soft tissue sarcoma in 9 cases (20%), histiocytic sarcoma in 2 cases (4%), history of severe trauma or nonfunctional limb in 2 cases (4%), mast cell tumour in 2 cases (4%), peripheral nerve sheath tumour in 1 case (2%), intramedullary sarcoma in 1 case (2%), scar revision with no neoplasia noted histologically in 2 cases (4%), severe synovitis in 1 case (2%), osseous haematoma in 1 case (2%), monostotic osseous lesion without histology submitted in 2 cases (4%), osseous plasma cell tumour in 1 case (2%), and peripheral nerve sheath tumour of brachial plexus in 1 case (2%). The most common reason for limb amputation in the CG was also an aggressive osseous lesion located in the peripheral limb with osteosarcoma being the most common disease process which occurred in 27 of 44 cases (61%). Other disease processes included soft tissue sarcoma in 4 cases (9%), mast cell tumour in 3 cases (7%), chondrosarcoma in 2 cases (5%), histiocytic sarcoma in 2 cases (5%), history of severe trauma or nonfunctional limb in 2 cases (5%), haemangiosarcoma in 1 case (2%), fibrosarcoma in 1 case (2%), malignant melanoma in 1 case (2%), adenocarcinoma in 1 case (2%), and cutaneous adenocarcinoma in 1 case (2%).

For the LBG, the typical postoperative analgesic protocol consisted either of intermittent boluses of an analgesic medication (hydromorphone) or continuous rate infusion of an analgesic (fentanyl, ketamine, both). Oral analgesic medications included a combination of a nonsteroidal anti‐inflammatory (meloxicam, Metacam, Boehringer Ingelheim, Germany; carprofen, Rimadyl, Zoetis, Parsippany‐Troy Hills, NJ; gapiprant, Galliprant, Aratana Therapeutics, Leawood, KS), opioid (tramadol, Tramadol, Sun Pharmaceuticals, Princeton, NJ; codeine, West Ward Pharmaceuticals Corp, Eatontown, MJ), and Gabapentin (Ascened Laboratories, Parsippany, NJ).

For the CG, the typical immediate postoperative analgesic protocol consisted of continuous rate infusion of an analgesic (fentanyl, hydromorphone, ketamine, methadone) either alone or in combination with intermittent opioid administration (buprenorphine, hydromorphone), and wound soaker catheters in select patients. A similar oral analgesic protocol was administered to the CG as the LBG group.

There were a higher number of patients who received an epidural within the CG (*n* = 12) compared to the LBG (*n* = 1). Patients in the control group were kept on continuous rate infusions of analgesic medication for a higher average length in hours compared to the liposomal bupivacaine group that was statistically significant (*p* = 0.0001). The number of continuous rate infusions was also statistically higher in the control group compared to the liposomal bupivacaine group (*p* = 0.0007).

Other parameters that were evaluated included the length in time from surgery to discharge between the groups which was statistically higher in the control group (average 24 h, range of 14 to 68 h) compared to the liposomal bupivacaine group (average 21 h with range 14 to 46 h) (*p* = 0.025). The first time to alimentation was also found to be statistically longer in the control group with an average of 13 h (range 6 to 27 h) compared to 8.5 h (range 2 to 27 h) in the liposomal bupivacaine group (*p* value = 0.0002). All patients in the liposomal bupivacaine group were noted to eat in hospital in contrast to 13 patients (30%) in the control group that did not eat in hospital prior to discharge but was not statistically significant.

Incisional complications were defined as notation of any of the following incisional changes noted in hospital or at any recheck examination postoperatively: seroma formation, incisional dehiscence, incisional signs of overt infection (incisional discharge, peri‐incisional swelling with associated pain, positive bacterial culture), incisional dehiscence, or any combination thereof). Within the control group there were a total of 15 incidences of incisional complications (34%) compared to the liposomal bupivacaine group which consisted of six incidences of incisional complications (13%). Of the 15 patients with incisional complications in the control group, four required revisional surgery (9%) whereas none of the patients with incisional complications required revisional surgery in the LBG. There was no statistical significance in incisional complications when comparing the LBG compared to the control (*p* = 0.053). The typical intended time frame for first recheck was 10–14 days postoperatively in both groups. When comparing the actual total number of rechecks needed postoperatively, the CG had a wider range of number of rechecks needed (1 to 6 rechecks) compared to the LBG (1 recheck) which was statistically significant (*p* = 0.001).

## 4 DISCUSSION

Results of the present study reveal that there was no increase in postoperative incisional complications with use of LBS as a local incisional and peri‐neural peripheral block for limb amputation procedures compared to the internal control group. The rate of incisional complications in patients with use of LBS was 13%, which is comparable to previously reported incisional complication rates evaluated in dogs with limb amputation procedures of 12.8% (Lascelles & Kirkby Shaw, 2016). Although not statistically significant, the percentage of incisional complications observed in the LBG was more than halved when compared to the internal control group rate of 34%. Additionally, none of the incisional complications noted in the LBG required revisional surgery, whereas 9% of patients within the control group had to undergo revisional surgery.

LBS is labelled to provide locoregional postoperative analgesia for up to 72 h post administration at the site of injection (Robbins et al., 2015). Currently there are two veterinary studies showing LBS to be highly efficacious in local pain management in stifle surgery (Nocita, 2018; Reader et al., 2020). The half‐life of bupivacaine is 4.8 h compared to LBS which is 36.2 h (Raske et al., 2015). The higher incidence of incisional complications in this study control group could be theorised to be correlated with discomfort postoperatively after discharge from hospital. When evaluating postoperative communications performed as early as 24 h after discharge through the first recheck examination in our study population, it was noted that owners in the control group were noted to have an increase in reported active attempts to lick or chew at the incision. It could be extrapolated that liposomal bupivacaine, due to its longer length of action of up to 72 h could prevent attention to incision during the inflammatory phase of wound healing. Previous studies have shown that patients that have been provided adequate analgesia within the first three days postoperatively have lower pain scoring but do not differ after 72 h (Costa et al., 2019).

Opioid usage in the postoperative setting can be associated with numerous negative side effects, amongst which include decreased gastrointestinal motility (Reid et al., 2007; Mehendale & Yuan, 2006) and dysphoria (Lemke & Dawson, 2000; Russell et al., 1982). The use of liposomal bupivacaine has previously shown to decrease the amount of opioid treatments while in hospital (Nocita, 2018). These findings were also found in our study population as the number and length of hours of continuous rate infusions used were significantly lower in the liposomal bupivacaine group compared to the control. Recovery quality has been previously assessed in dogs using an opioid model consisting of fentanyl versus other nerve blockage (epidural, peripheral nerve block) which revealed a significant worsening of recovery quality in those in the opioid group (Garofalo et al., 2012). A set of patients in the liposomal bupivacaine group were managed without continuous rate infusions consisting of opioids, with intermittent opioid administration used postoperatively. Many of these patients only received one dose immediately postoperatively with quick transition to oral analgesics. This could account for the decrease in undesirable opioid effects within the liposomal group, which could account for the quicker time to first alimentation and discharge compared to the control group.

The data revealing time to first alimentation further supports this as the control group was statistically noted to have their first alimentation much later postoperatively (average 15.3 h) than the liposomal bupivacaine group (average 9.6 h) or did not eat at all in hospital (*n* = 13 CG; *n* = 2 LBG).

Inherent limitations were present due to the retrospective nature of this study. The lack of standardisation of analgesic medication and reasons for selection of specific analgesic protocols was not specifically relayed in clinical records and suspected surgeon dependent.

The most significant and major limitation was that no statements could be made on efficacy as pain scoring was only present in 32% of patients in the control group and could not be directly compared with the liposomal bupivacaine group. This discrepancy reflects changes in hospital policy with implementation of consistent pain scoring in postoperative patients during the course of the study. When reviewing pain scores for the liposomal bupivacaine group using the Colorado State University Canine Acute Pain Assessment scoring system (Romano et al., 2016), all recorded values were below the threshold for re‐evaluation for rescue analgesia of ≥ 2 signifying mild to moderate pain (Romano et al., 2016). In order to comment on efficacy however, a prospective, blinded, clinical trial would be required to fully evaluate LBS efficacy on pain response using pain scoring.

From the results of this study, we can conclude that the extra‐label administration of LBS was well‐tolerated and effective in dogs undergoing limb amputation. LBS usage did not increase incisional complication rates and its use allowed for a generalised decrease in opioid usage and quicker time to discharge. To the authors knowledge this is the first published used of subepineural liposomal bupivacaine in amputations. A separate prospective, blinded, clinical trial would be required to fully evaluate LBS efficacy on pain response using pain scoring. Although not to be used as a sole analgesic modality, there are many potential uses for LBS as a local incisional analgesic that warrants further study.

## AUTHOR CONTRIBUTIONS

Ashley S Villatoro: data curation; writing – original draft; writing – review & editing. Holly A Phelps: conceptualisation; methodology; supervision; validation; writing – review & editing. Justin B Ganjei: conceptualisation; methodology; supervision; validation; writing – review & editing.

## CONFLICT OF INTEREST STATEMENT

The authors declare no conflict of interest related to this report.

## FUNDING INFORMATION

The authors received no financial support for the research, authorship, and/or publication of this article.

### ETHICS STATEMENT

The authors confirm that the ethical policies of the journal, as noted on the journal's author guidelines page, have been adhered to and the appropriate ethical review committee approval has been received. The US National Research Council's guidelines for the Care and Use of Laboratory Animals were followed.

### PEER REVIEW

The peer review history for this article is available at https://www.webofscience.com/api/gateway/wos/peer‐review/10.1002/vms3.1159.

## Data Availability

The data that support the findings of this study are available from the corresponding author upon reasonable request.
